# Monoclonal Antibodies in Pregnancy and Breastfeeding in Patients with Multiple Sclerosis: A Review and an Updated Clinical Guide

**DOI:** 10.3390/ph16050770

**Published:** 2023-05-21

**Authors:** Panagiotis Gklinos, Ruth Dobson

**Affiliations:** 1 Department of Neurology, Aeginition University Hospital, National and Kapodistrian University of Athens, 11528 Athens, Greece; 2 Preventive Neurology Unit, Wolfson Institute of Population Health, QMUL, London EC1M 6BQ, UK; ruth.dobson@qmul.ac.uk; 3 Department of Neurology, Royal London Hospital, Barts Health NHS Trust, London E1 1FR, UK

**Keywords:** multiple sclerosis, monoclonal antibodies, pregnancy, breastfeeding, disease-modifying therapies, review

## Abstract

The use of high-efficacy disease-modifying therapies (DMTs) early in the course of multiple sclerosis (MS) has been shown to improve clinical outcomes and is becoming an increasingly popular treatment strategy. As a result, monoclonal antibodies, including natalizumab, alemtuzumab, ocrelizumab, ofatumumab, and ublituximab, are frequently used for the treatment of MS in women of childbearing age. To date, only limited evidence is available on the use of these DMTs in pregnancy. We aim to provide an updated overview of the mechanisms of action, risks of exposure and treatment withdrawal, and pre-conception counseling and management during pregnancy and post-partum of monoclonal antibodies in women with MS. Discussing treatment options and family planning with women of childbearing age is essential before commencing a DMT in order to make the most suitable choice for each individual patient.

## 1. Introduction

Multiple sclerosis (MS) is a chronic inflammatory, demyelinating, and neurodegenerative disease of the central nervous system (CNS) which is often diagnosed in young women of childbearing age [1]. As many women diagnosed with MS will not have completed their family at the time of diagnosis, the proactive discussion of treatment options and the associated risks and benefits regarding pregnancy and breastfeeding is of high importance for many patients. These discussions need to be based on up-to-date evidence and, given the current rapidity of knowledge generation, should be regularly revisited. Current treatment paradigms increasingly highlight the importance of early and high-efficacy treatment to control disease activity and prevent subsequent disability [2,3,4,5]. Monoclonal antibodies, including natalizumab, alemtuzumab, ocrelizumab, and ofatumumab, represent an increasingly used class of high-efficacy disease-modifying treatments (DMTs).

Although there are a few studies suggesting that maternal MS is associated with an increased risk of adverse pregnancy or neonatal outcomes, it is well established that it does not affect fertility, nor does it increase the risk of miscarriage [6,7,8,9]. However, limited evidence on the relative safety of DMT in pregnancy for both mother and baby has led many women to either interrupt DMT or delay treatment initiation until after they have completed their families. Moreover, the regulatory guidelines on the use of many treatments during pregnancy and breastfeeding are highly restrictive. Real-world data from DMT exposures during pregnancy can take many years to collate due to safety concerns. Alongside this, achieving regulatory change can take decades, as has been the case with interferon-beta preparations. However, deferring treatment due to family planning concerns, particularly in cases where conception takes longer than anticipated, puts many women at risk of developing irreversible neurological damage as it is now well established that neurodegeneration starts early in the course of the disease [10].

Another issue of high relevance is breastfeeding. Many women have previously been advised to forego breastfeeding in order to recommence DMT, although the protective effect of breastfeeding on both the mother and the child is well described. Studies on the breastmilk transfer of monoclonal antibodies have been limited to date, and even where they exist, they have failed to translate into widespread clinical guidance [11]. Whilst breastfeeding likely reduces post-partum relapse risk, this moderate effect may not be sufficient to prevent relapses, particularly in women with highly active disease [12].

Family planning for people living with MS is thus a complex issue that should be addressed holistically in order that an optimal balance can be achieved. Assessing the risks of delaying or stopping treatment vs. the risk of fetal exposure to a DMT is crucial. A personalized approach based on both patient characteristics and the DMT mechanism and safety data is needed. Factors such as clinical and imaging characteristics, in addition to patient preference and attitude towards balancing risks, need to be taken into consideration when deciding on a treatment approach.

Hormonal changes during pregnancy, including an increase in estrogens and progesterone, have immunomodulatory effects on women and provide relative protection against disease activity during pregnancy [13]. Overall, in untreated cohorts, the annualized relapse rate (ARR) falls progressively during pregnancy before increasing in the postpartum period [14]. In more recent cohorts, the ARR has been shown to fall during the third trimester of pregnancy to as low as 0.2 relapses/person/year compared to 0.7 relapses/person/year during the year before conception [15,16]. However, this protection afforded by pregnancy might not be enough to control the disease in people with highly active disease, making the administration of high-efficacy DMTs potentially necessary during pregnancy. On the other hand, dual immunosuppression during pregnancy (hormonal changes and DMT) needs to be taken into consideration when deciding on a treatment plan, as it can lead to an increased risk of maternal infections. Therefore, in mild cases with minimal inflammatory activity before pregnancy, the protection afforded by pregnancy might be considered adequate by some neurologists.

However, treatment suspension or cessation, particularly of some of the highest-efficacy immunosequestering DMTs, such as natalizumab or fingolimod, has been associated with rebound disease activity, which can potentially cause catastrophic relapses [17]. Therefore, neurologists need to be extremely cautious when deciding to stop or suspend such drugs. In addition, in all people with MS, the absence of a clinical relapse during or subsequent to pregnancy does not necessarily equal complete control of disease activity. Studies have shown that more than half of people without clinical relapses will have radiological evidence of disease activity in the first year post-partum [18,19].

Furthermore, little evidence exists on the use of assisted reproductive technology and its association with disease activity and mAbs use. People with MS may seek fertility treatment (FT). Although there are studies suggesting that FT may increase disease activity, more recent and larger cohort studies failed to demonstrate the same results [20,21,22,23,24,25,26,27]. A large multicenter study included patients who had diverse FT, of whom 43% were on DMTs [27]. MAbs were associated with a reduced risk of relapse; however, pregnancy outcomes were not reported. More studies that investigate the association of DMT use with pregnancy outcomes in patients with FT are needed. In this review, we aim to assess the available data and provide an updated and comprehensive overview of the mechanisms of action, risks of exposure, and relevant pre-conception counseling, as well as management during pregnancy and post-partum of monoclonal antibody treatments in women with multiple sclerosis.

## 2. Monoclonal Antibodies and Family Planning in MS

Monoclonal antibodies (mAbs) have revolutionized the treatment of MS. Currently, there are five US Food and Drug Administration (FDA)- and four European Medicines Agency (EMA)-approved mAbs for the treatment of remitting–relapsing and progressive forms of MS [28]. These include natalizumab (Tysabri) for the treatment of relapsing–remitting MS (RRMS), which was first approved in 2004, then suspended in 2005 due to three cases of progressive multifocal leukoencephalopathy (PML), and re-introduced in 2006; alemtuzumab (Lemtrada) for RRMS (2013); ocrelizumab (Ocrevus) for both RRMS and progressive MS (2017); and ofatumumab (Kesimpta) for RRMS (2021). Recently, ublituximab (Briumvi) was approved by the FDA for the treatment of clinically isolated syndrome (CIS), RRMS, and active secondary progressive MS. Finally, rituximab (MabThera) is widely used off-label for both relapsing and progressive MS. MAbs are immunoglobulins that can be transferred to the fetus through the placenta. Active transport of maternal immunoglobulins across the placenta starts after the end of the first trimester. [29]. Fetal IgG is only 5–10% of the maternal IgG concentration between 17 and 22 gestational weeks; however, it increases exponentially during the second and third trimesters and exceeds or reaches a similar level to that in the maternal circulation at term; this is particularly the case with the IgG1 and IgG4 subclasses, which are those used in MS [30,31]. The minimal transplacental IgG transfer during the first trimester, along with each mAb’s half-life and mechanism of action, is particularly relevant when determining the optimal timing for mAb administration during pregnancy and in informing clinical decisions.

### 2.1. Natalizumab

Natalizumab, a humanized IgG4 mAb, acts as a selective adhesion molecule inhibitor and binds to the α4β1 and α4β7 integrins, which are highly expressed on the surface of lymphocytes and monocytes. It blocks their interaction with vascular cell adhesion molecule-1 (VCAM-1) and, through this mechanism, prevents leukocyte transmigration across the blood–brain barrier (BBB) to the brain parenchyma, controlling disease activity. Natalizumab is used for rapidly evolving severe MS (RES-MS) or in patients with breakthrough disease despite being treated adequately with another DMT. It is among the most effective DMTs, achieving high rates of suppression of focal inflammatory disease activity and the slowing of disability progression [32,33,34,35].

The lack of association of natalizumab treatment with congenital anomalies, with no increase in the rate of miscarriages seen across more than 500 natalizumab-exposed pregnancies [36,37], is relatively reassuring. This, combined with the risk to the mother from treatment discontinuation, means that an increasingly standard approach is to continue natalizumab treatment during pregnancy, albeit with extended dosage intervals. However, both the FDA and EMA advise discontinuing natalizumab once pregnancy is confirmed. The use of natalizumab during the third trimester of pregnancy has been linked to mild to moderate hematologic abnormalities in infants, including anemia, thrombocytopenia, and leukocytosis [38]. All cases in this series were self-limited within 4 months after birth and required no special treatment.

Women who discontinue natalizumab either before conception or during the first trimester of pregnancy are at substantial risk of rebound relapses 12–16 weeks after treatment discontinuation [39]. These relapses can be catastrophic, requiring high doses of steroids, plasma exchange (PLEX), or intravenous immunoglobulin (IVIG), resulting in a significant increase in disability [39,40]. A cohort study from the German MS Pregnancy Registry showed that women who stopped natalizumab after the first trimester had a lower relapse rate during pregnancy and post-partum compared to those who stopped it before the first trimester (5.9% vs. 32.4% during pregnancy and 22.8 vs. 49.7% post-partum, respectively) [41]. Pregnancy outcomes including pre-term birth or congenital abnormalities did not differ between the two groups. However, hematological abnormalities were observed in 53.8% of the newborns of women treated with natalizumab after the first trimester, with anemia being more common than thrombocytopenia [42]. Importantly, the closer to birth the exposure to natalizumab, the higher the risk of hematological abnormalities.

Based on placental transfer data and keeping in mind that most patients on natalizumab have either rapidly evolving severe MS or active disease despite being on another DMT, the current preferred practice includes continuing infusions until 30–34 weeks of gestation in extended interval dosing (6 or 8 weeks). Treatment should ideally be restarted 1–2 weeks after birth, while trying to not leave the patient untreated for more than 8–10 weeks [43]. Whilst natalizumab was originally licensed to be administered on a 4-weekly basis, recent data have shown that extended interval dosing is almost equally effective in preventing disease activity [44]. Whilst stopping natalizumab during the second trimester in order to minimize fetal exposure may be considered, this poses a greater risk of rebound disease activity during pregnancy. An alternative would be to switch to an immunodepleting DMT, such as ocrelizumab, prior to conception.

When neonates are potentially exposed to natalizumab during pregnancy, they should be checked for hematological abnormalities at birth. All live vaccinations should be avoided in infants exposed via transplacental transfer during the third trimester. In terms of breastfeeding, although there is some transfer, the concentration of natalizumab in breastmilk remains low. Currently, there are no signs that exposure to breastmilk increases the risk of hematological abnormalities in the child since systemic absorption by the baby is very unlikely. Experts recommend continuing natalizumab while breastfeeding given the benefits of both breastfeeding and natalizumab [45].

### 2.2. Alemtuzumab

Alemtuzumab is an IgG1 humanized monoclonal antibody which was licensed in 2013. It is a non-selective immune reconstitution therapy (IRT) and is considered one of the most potent treatments for MS. It is indicated for patients with RES-MS or with highly active disease despite a full and adequate course of treatment with at least one DMT. It is directed against the cell surface glycoprotein CD52, which is highly expressed on T (CD3+) and B (CD19+) lymphocytes and acts via antibody-dependent cellular cytolysis and complement-mediated lysis after binding to T and B cells. Through this mechanism, it causes severe immune depletion, followed by subsequent repopulation.

Alemtuzumab is administered by intravenous infusion for two initial treatment courses. It is not recommended for use during pregnancy or breastfeeding. The FDA and EMA advise avoiding pregnancy for at least 4 months after the last infusion. However, serum concentrations of alemtuzumab are very low or even undetectable 30 days after an infusion [35].

Treatment with alemtuzumab has been linked with a subsequent increased risk of immune-mediated conditions, which can be life threatening for the mother and the fetus. These include immune thrombocytopenic purpura (ITP), seen in 1% of treated patients, and autoimmune thyroid disorders in up to 40% [46]. Uncontrolled thyroid disease poses substantial risks for both the mother and the fetus and should be actively treated in pregnancy due to its association with low birth weight, pre-term birth, preeclampsia, and long-term neurocognitive impairment. Monthly blood monitoring with monthly thyroid function tests during pregnancy are crucial. Furthermore, placental transfer of anti-thyroid antibodies to the developing fetus can cause neonatal Grave’s disease even in euthyroid women; hence, close monitoring during pregnancy is particularly recommended. Obstetricians should also be aware of the potential risks.

Oh et al. studied pregnancy outcomes in the women who took part in the clinical trials of alemtuzumab [47]. Whilst there was an increased risk of spontaneous abortion in women treated with alemtuzumab, this was associated with older age (37% in women >35 years old vs. 15% in women <35 years old). Noticeably, the risk of spontaneous abortion was not increased in women who conceived in less than 4 months compared to those who conceived more than 4 months after the last alemtuzumab infusion, and neither was it different between women who developed a thyroid disorder and those who did not [47].

Given the widespread expression of CD52, MS specialists recommend delaying conception for 4 months after the last alemtuzumab infusion; however, if unplanned conception occurs sooner, women can be relatively reassured. Its low concentration a month after infusion, along with the minimal IgG transfer across the placenta during the first trimester, make its transfer to the fetus and its consequences quite unlikely. All live vaccines should be avoided in the mother and only administered after the exclusion of hematological abnormalities in potentially exposed neonates.

There are no data on the transfer of alemtuzumab to human breast milk. Its concentration in human breastmilk is presumably low due to its high molecular weight (>145 kDa), though detectable compared to the other mAbs. Based on that, MS experts recommend that breastfeeding can start earlier than 4 months after the last infusion. On the other hand, the EMA recommends waiting at least 4 months after the last infusion before breastfeeding.

### 2.3. Ocrelizumab

Ocrelizumab is a humanized monoclonal antibody, which is indicated for the treatment of active RRMS or active primary progressive multiple sclerosis (PPMS). It is administered by intravenous infusions every 6 months and acts by binding to CD20-expressing B lymphocytes and selectively depleting them through antibody-dependent cellular phagocytosis (ADCP), antibody-dependent cellular cytotoxicity (ADCC), complement-dependent cytotoxicity (CDC), and apoptosis. Although administered by 6-monthly infusions, the data from the phase II trials showed that the median repletion time for CD19+ B cells to either baseline or a lower normal range was 72 weeks (range 27–175 weeks) [48]. Increasing evidence on the potential utility of extending or adapting the ocrelizumab dosing interval according to the CD19+ count during the COVID-19 pandemic has led to considerations of the use of extended dose intervals for pregnancy purposes [49,50].

Ocrelizumab is an IgG1 monoclonal antibody; hence, it is transferred through the placenta to the fetus. Its mean half-life is 26 days; hence, it would normally take 18.5 weeks to completely clear from the circulation, as drugs are considered to require five half-lives to fully clear. Therefore, even if ocrelizumab was administered just before the time of conception, by the time placental transfer started at around 17–22 weeks of gestation it would have fully cleared; hence, fetal exposure would be minimal.

The largest registry of ocrelizumab exposure to date included 2020 cumulative MS pregnancies, of which 705 (34.9%) had in utero exposure (207 in the 1st trimester); 433 (21.4%) had no in utero exposure; and 882 (43.7%) had unknown exposure [41]. Of 1498 prospectively reported pregnancies, 596 (39.8%) had known outcomes: 471 (79.0%) live births (57.1% full-term, 10.0% pre-term, 32.9% unknown gestational week), 0.8% of whom had major congenital abnormalities; 11 (1.8%) ectopic pregnancies; 46 (7.7%) therapeutic/elective abortions; 67 (11.2%) spontaneous abortions; and 1 (0.2%) stillbirth. Of 532 prospectively reported pregnancies exposed in utero, 286 had known outcomes: 225 (78.7%) live births; 4 (1.4%) ectopic pregnancies; 33 (11.5%) therapeutic/elective abortions; 23 (8.0%) spontaneous abortions; and 1 (0.3%) stillbirth [51].

A further study included 88 pregnancies in women with neuroinflammatory disorders treated with ocrelizumab or rituximab, with the data not disaggregated by drug [52]. Rituximab is also an anti-CD20 monoclonal antibody, with a similar mechanism of action and mean half-life to that of ocrelizumab; this is discussed in more detail in a later section. In this study, of the 88 pregnancies, 10 were exposed more than 6 months before conception, 64 within 6 months of conception, and 14 after their last menstrual period. There were 67 known pregnancy outcomes. Five spontaneous miscarriages (7.4%) and nine pre-term births (15%) were reported—and these were significantly associated with exposure after the last menstrual period (45.5% vs. 9.8% *p* = 0.019). Two congenital abnormalities (ventricular septal defect and atrial septal defect with pulmonary stenosis) were also reported—both cases with exposure after the last menstrual period.

Out of the 14 neonates exposed to ocrelizumab or rituximab with available B-cell counts, there were 4 neonates with borderline counts. One of them was exposed during pregnancy, and in the other three cases, the last infusion had been administered 0–12 months before the last menstrual period (one case > 6 months and two cases < 6 months). One neonate was B-cell depleted, though in this case the mother was treated with azathioprine until the 7th week of gestation and with rituximab 141 days after the last menstrual period [52]. A case report included a woman with RRMS who was switched from natalizumab to ocrelizumab during pregnancy due to a high JCV antibody index and high disease activity; the woman had an ocrelizumab infusion at the 19th week of gestation. There was no B-cell depletion in the infant; neurological examination was normal at 3 and 17 months, and all developmental milestones were met [53]. Another study collected and analyzed data from the German MS Pregnancy Registry from 44 infants exposed to either ocrelizumab or rituximab (35 ocrelizumab and 6 rituximab) before/during pregnancy and lactation or lactation only [54]. Most of the postnatal B-cell counts were within normal range, and the two cases of complete B-cell depletion following second or third trimester exposure repopulated in a 2-month follow-up. Importantly, no congenital abnormalities during the first year of life were reported.

Exposure to ocrelizumab through lactation has not been reported to impact infant B-cell counts [54,55]. Similar results were reported from another study, which included 57 women exposed to either ocrelizumab or rituximab while breastfeeding [55]. The breastmilk concentration of both ocrelizumab and rituximab was low, and the median relative infant dose (RID) was 0.16% for ocrelizumab and 0.04% for rituximab, both well below the usually acceptable threshold of 10%. All infants had normal growth and development with no unexpected severe infections or other abnormalities.

Based on these data, MS specialists recommend either that patients can start trying to conceive without delay following an infusion or that they could wait 3 months after the last infusion for a more conservative and safer approach. Switching to extended interval dosing by measuring CD19+ B-cell counts has been shown to maintain treatment efficacy, while it increases the unexposed period and allows more time for conception in cases where it takes longer than anticipated. On the other hand, the FDA advises delaying conception for at least 6 months after an infusion, and the EMA advises waiting for at least 12 months. However, these recommendations are very conservative and partially explained by the limited data at the time of ocrelizumab’s approval.

When it is clinically indicated, e.g., in extremely active cases, ocrelizumab can be administered even during pregnancy, although it is not licensed. Where treatment is offered during pregnancy, people should be warned about the increased risk of neonatal B-cell depletion. Live vaccines should be deferred for as long as the effect of treatment on the immune status of the infant remains possible, i.e., until the B-cell levels have recovered. It is therefore recommended to measure the CD19+ B-cell level in neonates and infants prior to vaccination [45]. Although ocrelizumab treatment during breastfeeding is not approved, the data show that it is totally safe and that the concentration of the drug in the breastmilk is very low. Moreover, its low oral bioavailability would also decrease infant absorption. Therefore, ocrelizumab can be given during breastfeeding.

### 2.4. Ofatumumab

Ofatumumab is a fully human, anti-CD20 monoclonal immunoglobulin G1 (IgG1) antibody, which is administered by subcutaneous injection every month. The binding of ofatumumab to CD20 induces lysis of CD20+ B cells in both high- and low-CD20-expressing cells.

Ofatumumab is not licensed for use during pregnancy. The FDA and EMA recommend avoiding pregnancy for at least 6 months following the last injection. Data on the effect of ofatumumab on pregnancy outcomes are limited in humans, mainly because of its relative novelty outside highly specific indications. Pregnancy outcomes were collected from the clinical trials and the post-marketing surveillance program and included 32 pregnancies, of which 4 were exposed within 6 months and 12 had first-trimester exposure [56]. Of the 23 known outcomes, there were 11 live births, 6 miscarriages, and 6 elective terminations. There were no reports of B-cell depletion or immunoglobulin/hematological abnormalities, and no serious infections were specified. In terms of breastfeeding, a study on the use of anti-CD20 mAbs included two women who were on ofatumumab treatment while breastfeeding [54]. There was no effect on the B-cell counts in the infants who were exposed to the drug during lactation.

Currently, experts recommend either stopping injections when starting to try to conceive or even when pregnancy occurs. Alternatively, some patients with very active disease could be switched to ocrelizumab before starting to try to conceive; this would have long-lasting effects and most probably would protect them for the following 9–12 months.

All live vaccines must be avoided by the mother and only administered after the exclusion of B-cell depletion in exposed neonates. Ofatumumab has a high molecular weight; hence, its concentration in the breastmilk is very low or undetectable. Therefore, it is considered safe to breastfeed while on the treatment.

### 2.5. Ublituximab

The most recent addition in the arsenal of anti-CD20 mAbs, ublituximab, was approved by the FDA in December 2022 and is currently in the process of obtaining EMA approval. Ublituximab is a chimeric, glycoengineered, anti-CD20 mAb, that targets a unique epitope on CD20+ B cells. It has a low-fucose fragment crystallizable (Fc) region, allowing efficient B-cell depletion in much lower doses via antibody-dependent cellular cytotoxicity [57,58].

Due to its very recent approval, there are currently no data on the use of ublituximab in pregnancy and/or breastfeeding. However, it is expected to have similar characteristics to the other anti-CD20 mAbs. Its mean half-life is 22 days, which means that it would have fully cleared by the time transplancental transfer started (17–22 weeks of gestation), and therefore, fetal exposure would be minimal even if the last infusion had been administered just before conception. Although there are not enough human data yet, transient lymphocytopenia and B-cell depletion can be anticipated in the case that the fetus is accidentally exposed during pregnancy. The risk of major birth defects, miscarriages, or adverse maternal or fetal outcomes is currently unknown.

Although the FDA recommends administering the last infusion at least 6 months before trying to conceive, it is expected that no harm would be caused to the fetus if the last dose was given closer to or even just before conception, as with the other anti-CD20 treatments. However, more data from human studies are needed to confirm this.

In terms of breastfeeding, it is expected that ublituximab would be excreted in the breastmilk; however, its concentration should be very low and similar to that of ofatumumab as it has almost the same molecular weight (ublituximab-147 kDa, ofatumumab-145 kDa). Consequently, its absorption should be minimal. However, more data from human studies that would measure its concentration in the breastmilk are required.

### 2.6. Rituximab

Although rituximab is not a licensed DMT for MS, it is widely used off-label. It is a chimeric IgG1 monoclonal antibody that binds to the CD-20 antigen on the surface of B cells and induces B-cell lysis via complement-dependent cytotoxicity (CDC), which results from C1q binding, and antibody-dependent cellular cytotoxicity (ADCC), which is mediated by one or more of the Fcγ receptors on the surface of granulocytes, macrophages, and NK cells. Its binding to CD-20 antigen on B cells has also been shown to induce cell death via apoptosis.

The FDA and EMA recommend stopping rituximab infusions 12 months before conception. Studies of more than 200 pregnancies have shown that it was not associated with congenital abnormalities or adverse pregnancy outcomes, even in pregnancies that occurred within 6 months of the last infusion [52,59,60]. However, one of the studies reported a high rate of spontaneous abortions (27%), which could possibly be attributed to the high rate of pre-existing infertility in half of the cases [59].

Data from non-neurological conditions can also be used to assess rituximab’s safety in pregnancy. A systematic review included 102 pregnancies with exposure either within 6 months before conception or during pregnancy [60]. Of the 104 known outcomes, there were 73 live births, 1 stillbirth, 14 miscarriages, and 15 elective abortions. Another study included 21 women who had exposure either within 6 months or during pregnancy [61]. Of the 19 known outcomes, two women had an infusion shortly after conception and one continued treatment until the second trimester. No miscarriages, stillbirths, or serious infections were reported. The only adverse outcome included an infant born with multiple hemangiomas. Finally, another study of rituximab in the treatment of pemphigus included 19 pregnancies with exposure to rituximab. Of the 19 known outcomes, there were 17 (89%) live births, 1 termination (5%), and 1 spontaneous abortion (5%). There was also one case of neonatal sepsis with good recovery. [62].

As with ocrelizumab, and based on this evidence, the current experts’ practice is to stop treatment either 3 months before or by the time of conception. All live vaccines should be avoided in the mother and only administered after the exclusion of B-cell depletion in exposed neonates. Breastfeeding is considered safe since rituximab’s concentration in the breastmilk is very low. The RID has been found to be only 0.04%, which is acceptable [54].

Table 1 summarizes monoclonal antibodies’ characteristics and their use in pregnancy and breastfeeding.

## 3. Discussion

MS affects many patients of childbearing potential. Early family planning and counselling in this group are essential. Counseling on mAbs use during pregnancy and breastfeeding should start before the patient stops contraception. Monoclonal antibodies have been increasingly used over the past years with excellent control of focal inflammatory activity, as well as slowing of disability progression. A personalized treatment strategy needs to be agreed with the patient before starting to try to conceive in order to preserve disease suppression alongside fetal safety. Current FDA and EMA recommendations are restrictive and conservative, which can lead to MS being undertreated, leaving patients unprotected for a long time, particularly in cases where conception takes months or even years. Although in most cases pregnancy is a time with decreased inflammatory activity, it is well known that activity increases post-partum and even during pregnancy in patients with very active disease or in patients previously treated with natalizumab or fingolimod (rebound activity). The risks of fetal exposure during pregnancy and the risks of treatment discontinuation or delay in the initiation of treatment must be balanced. A personalized treatment plan, based on clinical, imaging, and biochemical (levels of neurofilament) characteristics is key. Leaving a patient unprotected against disease activity for a long time increases the risk of relapses and disability accumulation, which in the long run might turn out to be detrimental.

**Figure 1:**
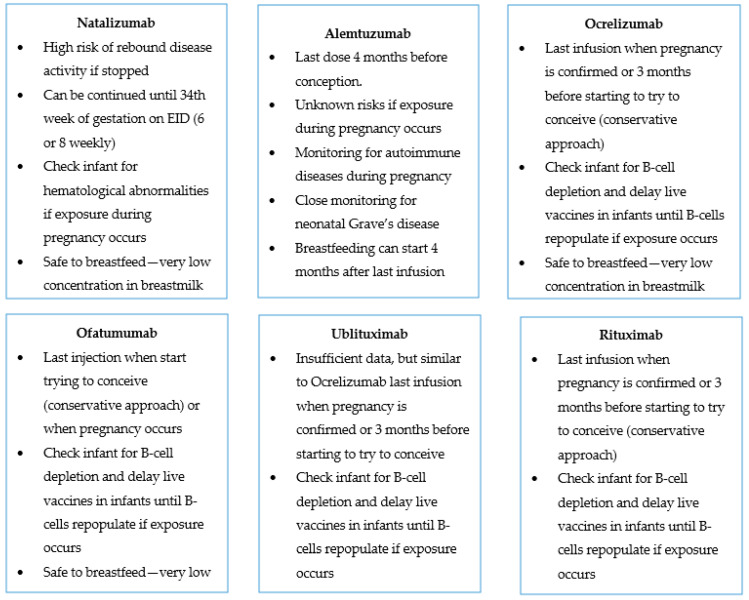
Summary of most important facts on the use of mAbs during pregnancy and breastfeeding. EID: extended interval dosing.

Based on the available data, MS specialists recommend continuing treatment with natalizumab until 30–34 weeks of gestation given the high risk of rebound activity if stopped earlier. Alemtuzumab should be administered at least 4 months before conception. Ocrelizumab and rituximab should not routinely be administered during pregnancy other than in the case of uncontrolled significant disease activity. In usual practice, the last infusion should occur before pregnancy occurs, ideally without prolonged drug-free intervals. Finally, ofatumumab should be stopped when stopping contraception or when pregnancy occurs in patients with high disease activity. Close monitoring of the infants is needed to exclude any hematological abnormalities, particularly if exposed during the last trimester of pregnancy. Figure 1 summarizes the key characteristics of each mAb regarding its use around pregnancy and breastfeeding. 

## 4. Conclusions

MS is a disease that does not affect fertility, nor increases the risk of congenital abnormalities. Its management during pregnancy can be challenging particularly in patients with active disease due to the risks of fetal exposure, however, emerging treatments and safety data allow for adequate control of the disease with essentially low to minimal risk for the fetus. Neurologists need to stay up to date in today’s era of rapidly evolving advances in this field and provide their patients with the latest available data on the use of monoclonal antibodies in patients planning to start a family.

Although monoclonal antibodies are increasingly used and MS is increasingly diagnosed in young women of childbearing potential, the data regarding mAbs use during pregnancy and during breastfeeding are still limited. Importantly, more high-quality, prospective studies on pregnancy outcomes and the use of mAbs during breastfeeding are needed to establish robust conclusions with regard to exposure and safety. Patients should not be advised to discontinue mAbs long before conception or delay treatment in the context of family planning as current evidence supports their use during pregnancy in a reasonable way when they are needed. On the other hand, the long-term effects of the use of mAbs during pregnancy are not clear and are yet to be fully elucidated. The risk of disease reactivation, either with disabling relapses post-partum or during pregnancy or with only signs of radiological activity, and the long-term consequences should be taken into consideration and discussed with patients when deciding whether and when to stop mAbs in the context of family planning. MS pregnancy registers need to be developed across the world and more studies need to be conducted in order to monitor the outcomes and to gather more data. Guidelines and recommendations need to be revised on a regular basis according to the emerging data.

## Figures and Tables

**Table 1:** Monoclonal antibodies in pregnancy and breastfeeding.

	Natalizumab	Alemtuzumab	Ocrelizumab	Ofatumumab	Ublituximab	Rituximab
**IgG subclass**	IgG4	IgG1	IgG1	IgG1	IgG1	IgG1
**Route of administration and dosing intervals**	IV *—4 weekly	IV *—2 courses 1 year apart	IV *—6 monthly	SC *—monthly	IV *—6 monthly	IV *—6 monthly
**Use in pregnancy**	Can be used	No	Not routinely	Not routinely	Not routinely	Not routinely
**Mechanism of** **action**	Anti-trafficking, binds to α4-integrin expressed on lymphocytes	Immune reconstitution therapy, anti-CD52	Anti-CD20, B-cell depletion	Anti-CD20, B-cell depletion	Anti-CD20, B-cell depletion	Anti-CD20, B-cell depletion
**Risk of** **rebound** **activity if stopped**	High	No	No	Unknown	Unknown	No
**Last dose**	Last infusion no later than 34 weeks of gestation.	Last infusion no later than 4 months before starting to try to conceive.	Last infusion 3 months before starting to try to conceive (conservative approach) or stopping when pregnancy is confirmed.	Last injection when starting to try to conceive (conservative approach) or stopping when pregnancy is confirmed.	Last infusion 3 months before starting to try to conceive (conservative approach) or stopping when pregnancy is confirmed.	Last infusion 3 months before starting to try to conceive (conservative approach) or stopping when pregnancy is confirmed.
**Risk of** **miscarriage**	No	Insufficient data	No	Not enough data	Not enough data	Probably not; there is a study with high rate of miscarriage, though it was attributed to pre-existing infertility
**Fetal exposure** **during pregnancy risks**	Hematological abnormalities	Unknown	Neonatal B-cell depletion	No data	No data	Neonatal B-cell depletion
**Other considerations**	-	Neonatal Grave’s disease	-	-	-	-
**Breastfeeding** **(≥2 weeks post-partum)**	Yes	Wait 4 months after last infusion	Yes	Yes	No data	Yes
**Vaccinations**	Avoid live vaccines in mother. Postpone in neonates exposed during third trimester.	Avoid live vaccines in mother. Exclude hematological abnormalities in neonates before live vaccines	Avoid live vaccines in mother. Exclude low B-cell counts in neonates before live vaccines	Avoid live vaccines in mother. Exclude low B-cell counts in neonates before live vaccines	Avoid live vaccines in mother. Exclude low B-cell counts in neonates before live vaccines	Avoid live vaccines in mother. Exclude low B-cell counts in neonates before live vaccines

* IV: intravenous, SC: subcutaneous.

## Data Availability

No data were created.
